# Perspectives of Nurses and Doulas on the Use of Information and Communication Technology in Intercultural Pediatric Care: Qualitative Pilot Study

**DOI:** 10.2196/16545

**Published:** 2020-03-17

**Authors:** Nataliya Berbyuk Lindström, Rocío Rodríguez Pozo

**Affiliations:** 1 Department of Applied IT University of Gothenburg Gothenburg Sweden

**Keywords:** child care, migrant mother, nurse, doula, smartphone, mobile phone, Google Translate, internet, mHealth, digital health, intercultural communication

## Abstract

**Background:**

Sweden is rapidly becoming an increasingly multicultural and digitalized society. Encounters between pediatric nurses and migrant mothers, who are often primary caregivers, are impeded by language problems and cultural differences. To support mothers, doulas, who are women having the same linguistic and cultural backgrounds, serve as cultural bridges in interactions with health care professionals. In addition, information and communication technology (ICT) can potentially be used to manage interactions owing to its accessibility.

**Objective:**

The objective of this study was to investigate the role of ICT in managing communicative challenges related to language problems and cultural differences in encounters with migrant mothers from the perspectives of Swedish pediatric nurses and doulas.

**Methods:**

Deep semistructured interviews with five pediatric nurses and four doulas from a migrant-dense urban area in western Sweden were audio recorded, transcribed, and analyzed using thematic content analysis.

**Results:**

The results showed that ICT contributes to mitigating communicative challenges in interactions by providing opportunities for nurses and migrant mothers to receive distance interpreting via telephones and to themselves interpret using language translation apps. Using images and films from the internet is especially beneficial while discussing complex and culturally sensitive issues to complement or substitute verbal messages. These findings suggest that ICT helps enable migrant mothers to play a more active role in interactions with health care professionals. This has important implications for their involvement in other areas, such as child care, language learning, and integration in Sweden.

**Conclusions:**

The findings of this study suggest that ICT can be a bridging tool between health care professionals and migrants. The advantages and disadvantages of translation tools should be discussed to ensure that quality communication occurs in health care interactions and that health information is accessible. This study also suggests the development of targeted multimodal digital support, including pictorial and video resources, for pediatric care services.

## Introduction

### Migration and Child Care in Sweden

At present, about 20% of the Swedish population is foreign-born [1]. Because of increased migration, intercultural encounters in pediatric care are becoming common. Many families of non-European background who migrate to Sweden with small children live in disadvantaged areas that are characterized by poor socioeconomic conditions [2,3]. Low income, insufficient Swedish language competence, and poor knowledge about health programs may have adverse impacts on the health of children in terms of mortality, morbidity, and injuries, and they could be determinants of physical and mental ill health in the future [4].

Being a parent and a migrant is a complex and challenging experience. Facing different health and social systems and lacking supporting social networks, such as relatives and friends, may result in psychological distress and depression [5]. This often causes migrants to avoid contact with locals in general and health care providers in particular, thereby deepening social exclusion [6,7]. As in many non-Western societies, mothers are the primary caregivers, and their contact and communication with pediatric nurses are essential for their health and the health of their children and families [8,9].

Child health care (CHC) centers led by a pediatric nurse or a district nurse offer free preventive health care to 99% of children in Sweden. Nurses assess child development, vaccines, nutrition issues, and parental health, including sleeping and breastfeeding, as well as social and family issues [10]. In meetings between migrants and health care staff, language problems are known to be a major barrier for communication, resulting in a lack of understanding, insecurity, and low engagement during interactions [11,12]. Although people without the ability to communicate in Swedish have a statutory right to interpreters [13,14], the use of interpreters is often challenging owing to patient refusal, lack of authorized interpreters, time shortage, etc [11,15-17]. Apart from language problems, cultural differences regarding beliefs, values, and practices often have a considerable impact on interactions in health care contexts [18-20]. Cultural competence and sensitivity when staff engage with migrant parents, as well as respectfulness, understanding, and cultural knowledge of the large ethnic minority groups living in the country are prerequisites for developing a trustful relationship and providing quality care in a multicultural society [8,21-23].

To support migrant parents’ interactions with individuals in the Swedish health care system, doula culture interpreters are available during pregnancy and birth and in the postpartum period. Doula (from Greek δούλα, doula, [pron. /ˈðula/], “servant woman”) is a migrant mother, who has Swedish knowledge and is willing to act as a “cultural bridge“ between mothers with the same cultural background and individuals in the Swedish health care system. The tasks of doulas include offering support during and after pregnancy, giving informal advice and information about health care services, and providing assistance during interactions with health care staff [24-26]. At this moment, 35 doulas are available in Gothenburg, and they are financially supported by the county council Region Västra Götaland (Western Sweden) [26,27].

### Digitalization and Migrant Care

Increasing digitalization in Europe, particularly in Sweden, is another barrier in terms of the digital skills gap that many low-skilled migrants must overcome when entering the host society [28-30]. Yet, from another perspective, information and communication technology (ICT), defined as a “diverse set of technological tools and resources used to transmit, store, create, share, or exchange information” [31], which includes computers, the internet, broadcasting, and telephony, provides integration support for developing contacts with locals [32], learning language and culture [33-35], and obtaining information about health care services [36].

With the purpose of mitigating linguistic barriers in communication between migrants with limited language competence and health care providers, mobile medical translation apps, such as American Canopy speak [37], Spanish Universal doctor [38], Australian CALD assist [39], and Swedish Care to Translate [40], provide translations, phonetic scripts, and audio recordings in minority languages for the different phases of consultation (eg, greeting and diagnosis). Although these types of apps are useful for communicating preset phrases, they are not able to replace professional interpreters [39,41]. To manage interactions with illiterate migrants, picture-based information and communication boards, partially available online, have been developed [42]. In addition, Google Translate, though criticized, is often used to manage language problems in intercultural interactions [32,43].

Currently, little is known about the use of ICT for managing communication between migrants with limited language competence and staff in pediatric contexts. More specifically, research on the role of technology for handling cultural differences in interactions is also limited [41,44]. This study explores the role of ICT in managing communicative challenges related to language and cultural barriers in interactions with migrant mothers from the perspectives of Swedish nurses and doulas.

## Methods

### Study Location

The study was conducted in a migrant-dense urban area in western Sweden. A qualitative inductive approach was chosen to identify patterns in data in an unprejudiced way [45]. Purposive sampling [46] was used for participant recruitment to obtain respondents with experience in communicating with migrant mothers.

### Recruitment

The author RRP contacted the chief nurses in three CHC centers in the chosen area of Gothenburg by email to inform them about the study. One center showed interest in participating in the project. The main reason for refusal by the other centers was time constraints.

Both authors received an invitation to present the study to nurses, who had experience working with migrant mothers, at a morning meeting. Five female Swedish nurses (aged 34-65 years), who were native Swedish speakers, agreed to participate in the interviews.

The author RRP also contacted the Doulas and Cultural Interpreters center via email and was invited to present the study to the doulas. Four doulas (aged 35-60 years) from Somalia, Iran, and Iraq, who had worked for 2-10 years as doulas, volunteered to participate.

### Data Collection

Semistructured interviews with five nurses and four doulas were conducted from February to May 2018. The interviews with nurses were conducted by both authors at the CHC center, whereas the author RRP conducted interviews with the doulas at the Doulas and Cultural Interpreters center. A semistructured interview guide for nurses and doulas was developed and piloted in five students, which resulted in minor changes (Table 1).

The interviews were audio recorded upon receiving written consent. Each interview lasted between 45 and 60 minutes. The total interview time was 417 minutes. The interviews focused on the experiences of doulas and nurses when communicating with migrant mothers and on the use of ICT for managing interactions.

**Table 1:** Interview guide.

Questions	Probes
What communicative problems influence your interaction with migrant mothers?	Word finding problems? Understanding problems? Cultural differences? Which ones?
How do you solve these problems?	By yourself? Getting help from interpreters? From mothers?
How is technology used in your interactions with migrant mothers to manage communicative problems?	The Internet? Stationary phones? Smartphones? Computers? Other tools? To manage language problems? To communicate culturally related issues?

The research was a part of the “Integration With Mobiles: Developing Language and Intercultural Communication Support for Integration of Newly Arrived Migrants” project approved by the Ethics Review Board of the Department of Applied Information Technology, University of Gothenburg, Gothenburg, Sweden (registration number: 538-17).

### Data Analysis

The interviews were transcribed verbatim in Swedish, translated into English by the author RRP, and checked against the original audio recording by the author NBL. Thematic content analysis was used for evaluation [47]. Data analysis involved an iterative process of listening to the interviews, reading the transcriptions, assigning codes, and finally determining patterns in the material. Both authors read the transcripts independently several times and assigned codes. Thereafter, the codes were sorted into different categories. The codes that had a high degree of agreement between the coders were discussed and sorted into subthemes. The subthemes were organized into overarching themes.

## Results

### Identified Themes

The following three broad themes were derived from the data in relation to the use of ICT to manage language problems and cultural differences in interactions: (1) using formal and informal distance interpreting; (2) using mobile translation apps; and (3) using images and films as a substitute for or complement to verbal messages.

### Using Formal and Informal Distance Interpreting

Nurses mentioned that many mothers they met on a daily basis had limited or no command of the Swedish language. A common view was that language problems were the most frequent challenge to overcome. Although professional interpreters were often available, in some situations, it was not possible to get them to come to the CHC center, and distance interpreting was arranged via stationary phones. Nurses acknowledged that this approach solved immediate communication needs, but they expressed skepticism about the quality of the interpreting service as follows:

The (phone) interpreter is unable to see what the children, nurse, and mother are doing in the room. It doesn't work so well. Especially when the child is older; there are so many things to do: to talk a little with her, to sit and draw with her here... the young sister screaming at the back. The interpreter misses a part...

Nurse #2

Lack of visual cues in combination with disturbances due to the presence of children and other family members was mentioned. Distance interpreting was also perceived as impersonal, which resulted in many mothers refusing this communication medium. Some interviewees argued that being unwilling to talk about health problems with interpreters owing to their perception as strangers and the fear that they would breach confidentiality was another reason for migrants avoiding professional interpreters. Both nurses and doulas reported that many mothers preferred to call their relatives, friends, or doulas using private smartphones and ask for mediating interactions rather than using professional interpreters. Mobile apps, such as WhatsApp and Viber, were commonly used. The following statement was made:

Many call their friends and their husbands who are at work and ask for help.

Nurse #1

Although nurses were critical about mothers involving informal interpreters, they had to accept the situation to avoid conflicts. The main concerns, apart from the quality of interpreting (often it was the older children in the family who acted as informal interpreters because they spoke better Swedish than their mothers, fathers, or mothers’ friends), were the lack of face-to-face contact and poor connection quality, which complicated understanding.

Doulas spoke about getting calls from mothers (ie, the mothers they were responsible for) and requests to interpret interactions with nurses, without prior notice. One doula commented as follows:

Sometimes we just get a call from the Child Health Center when they (a mother and a nurse) don't have an interpreter, and we interpret for them.

Doula #1

For doulas, an unplanned interpreting request was an additional task to perform, which led to frustration resulting from the blurred line between work as a doula and their private life. As mothers could call them on their private numbers and ask for help at any time, some doulas felt pressed to be constantly “connected” on their smartphones. All doulas commented on feeling bad about refusing to help mothers coming from the same country, as it could jeopardize their relationship. Concerns about not always being able to provide spontaneous support and being worried about not having enough knowledge to interpret appropriately were also expressed.

### Using Mobile Translation Apps

Participants mentioned migrant mothers refusing both formal and informal interpreting in meetings with nurses, which was motivated by having sufficient linguistic competence, as well as an unwillingness to involve anyone else in the interaction. Some mothers overestimated their Swedish proficiency, which required nurses to speak slower, use shorter simplified phrases, repeat their statements, and use gestures to illustrate what was meant. Drawing/writing on paper, writing the names of medicines, and using English or other languages was also mentioned. Concerning technology use, Google Translate was reported to be the most common (apart from Lexin, which is a Swedish online dictionary for Swedish and main minority languages in the country) and often the only translation app used. Nurses and doulas were positive about this translation app and mentioned encouraging mothers to “Google” and translate Swedish words into their native language. One doula said the following:

Sometimes I ask them (mothers) to Google translate, for instance the word mucus plug (into their language). I show that they can manage themselves (without my help).

Doula #3

In some cases, if longer text needed to be translated (eg, a website), nurses reported mothers “copying and pasting” it directly into Google. Talking about this issue, a nurse said the following:

I have parents who speak good Swedish, but when they need to write something, they use Google Translate. They scan the text and then you get the translation.

Nurse #3

### Using Images and Films as a Substitute for or Complement to Verbal Messages

Although Google Translate was primarily used to manage language problems, doulas and nurses reported showing images and videos from the internet on computer screens and mothers doing the same on their smartphones for managing language problems, as well as facilitating communication about cultural issues.

In situations where there was insufficient language or uncertainty about understanding, images from the internet served as a substitute for or complement to verbal communication to ensure understanding. A nurse commented as follows about using an image to illustrate baby food in a jar to complement the verbal message when she was unsure if mothers new in Sweden could understand her:

I can show the picture sometimes from my computer, so they understand what a prune is. I look for images of jars with beans and broccoli.

Nurse #2

Doulas and nurses were concerned about having limited time for interactions with mothers, which, in combination with language difficulties, resulted in stress and problems with allocating time for providing lengthy explanations concerning complex and sensitive issues. Many migrant mothers, especially those with less education, were perceived as having a more traditional perspective on their role as compared with native-born Swedish mothers. Being the primary and only responsible parent for child care resulted in a lack of time for learning Swedish and finding employment. All nurses commented that the CHC center, which was situated in a migrant-dense urban area, was often the only “window” for many mothers to the Swedish society. Not surprisingly, according to both doulas and nurses, many migrant mothers retained their cultural beliefs and values, which were often different from the Swedish cultural beliefs and values that were advocated by nurses. Thus, apart from time pressure and language difficulties, participants feared problems with understanding and mothers taking offence when Swedish cultural values regarding child care collided with the values from their countries of origin.

When talking about culture-sensitive issues in child care, both doulas and nurses reported using images and films from the internet to illustrate what they were trying to say, thereby complementing or substituting verbal messages. One of the most common cultural differences many migrant mothers experience in Sweden is related to nutrition, overweight, and obesity. Both doulas and nurses commented on the preferences of many mothers (eg, those from Somalia and Afghanistan) regarding the use of formula instead of breastfeeding or as a complement to breastfeeding, the addition of sugar to baby food, and the belief that a chubby baby was a sign of well-being, whereas there were differing views in Sweden. Participants reported that showing images of Swedish food from the internet on a computer screen was common when talking about healthy food habits. One nurse said the following:

You look for it (food) in Google and show not only the name but how it looks. When you're talking about porridge, for example, not everyone eats porridge as we do in Sweden. I Google and show how it looks. “This is a good porridge. There are others that have a lot of sugar. Avoid this kind!”

Nurse #4

The excerpt above shows the nurse using an image as a complement to the recommended porridge name. In addition, she also uses images of other products that contain more sugar, thereby making clear recommendations concerning limiting sugar intake and choosing the right foods.

In relation to weight problems, a doula mentioned using a projector in the center and showing mothers images about the changes to a woman’s body after delivery and breastfeeding, inspiring them to exercise for weight loss and go to the gym, which is an issue considered too sensitive to openly discuss. She said the following:

We show pictures. We have a projector. We illustrate how you should breastfeed, and we talk about parent education in Sweden, you know. How your body will go back to normal after delivery, going to the gym, etc.

Doula #1

Images are also used to help mothers understand more complex concepts, such as maternal-infant bonding. The participants mentioned that many migrant mothers consider bonding with babies in terms of keeping eye contact, playing, reading, and talking as being less important when compared with providing food and care. A doula commented as follows on attempting to show what skin-to-skin contact is using pictures from Google:

If I want to show what skin-to-skin contact is, then I Google it. I also ask them (the mothers) to Google. I show that they can do it themselves.

Doula #3

A nurse also mentioned regularly using a short film about safety available on the internet to both illustrate the risks at home and rules for child protection expected in Sweden. She said the following:

I have a good film on www.dinsakerhet.se (“yoursafety.se”), which is 4 minutes long because it shows burning wounds or a child climbing up on the sofa. In many countries, you do not have car seats. You have no bicycle helmets. You do not have child protection. We have to talk about it…

Nurse #2

This respondent stated that it was not easy to explain child protection measures, as many migrant mothers did not understand the difference between car seats for children of different ages and how to use bicycle helmets. As these measures are required by law in Sweden, the nurse felt obliged to inform migrant mothers about these measures. The short film about safety from the website was used to substitute explanations in order to save time. She also commented asking mothers to show the film to their partners and other family members. Another resource mentioned was www.1177.se (Vårdguiden–Health Care Guide), which is available in many languages and could be accessed during and after interactions. Both doulas and nurses complained about sometimes spending too much time on searching for suitable and good quality images and films on the internet (eg, YouTube). They also expressed a need for the development of multimedia resources that were targeted at the pediatric practice for managing intercultural interactions.

## Discussion

### Principal Findings

This qualitative pilot study explored the experiences of doulas and nurses using ICT to mitigate language problems and cultural differences in interactions with migrant mothers in an immigrant-dense urban area in Sweden.

The results of this study show that ICT contributes to mitigating communicative challenges in interactions by providing opportunities for nurses and migrant mothers to obtain distance interpreting via stationary phones and smartphones if interpreters are not physically present in the CHC center for different reasons [48]. While distance interpreting by professionals is preferred by nurses, migrant mothers prefer to call their friends, relatives, or doulas via private smartphones for informal interpreting [49]. In both cases, although distance interpreting has disadvantages, specifically in terms of sound quality due to poor connection (migrant mothers reported primarily using Voice over Internet Protocol apps, such as WhatsApp and Viber, which require an internet connection) and lack of visual messages [48], technology still helps to solve immediate communication problems. Interestingly, even though both WhatsApp and Viber provide opportunities for video calls, the respondents in this study did not mention using video calls, despite the fact that they can potentially contribute to communicating more visually [50] and possibly improving interpretation quality. The findings that migrant mothers use their smartphones and make active choices to contact informal interpreters indicate that mobile technology and the internet contribute to increased patient-centeredness and empowerment [51] by providing opportunities for solving communication challenges and enabling migrant mothers to take more responsibility for their own care and their children’s care. However, apart from corroborating the disadvantages of informal interpreting described earlier [52], this study indicates that informal interpreters (eg, husbands at work and doulas), who are perceived as a close, friendly, and supportive resource, are often contacted and expected to interpret without prior notice. The disturbance and pressure resulting from unplanned interpreting can additionally compromise the interpretation quality.

Another finding of this study was the extensive use of Google Translate for managing language problems in interactions, and it contributed to providing more opportunities for migrant mothers, nurses, and doulas to manage interactions themselves, without the assistance of a professional interpreter. Both nurses and doulas were positive about using Google Translate and reported asking mothers to “just Google,” believing that in this way, they encouraged them to become more involved in conversations. In accordance with the present results, previous studies have demonstrated that mobile translation and language apps are successful in relating language learning to a person’s physical context and are beneficial for informal learning of the language relevant to a specific context (pediatric encounters in this case) [33]. Although the fallacies and inaccuracies in automatic translations are well-known, especially for less common languages and for medical vocabulary [43,53], the use of Google Translate can potentially contribute to mothers acquiring the Swedish language and integrating into Swedish society.

While providing culturally competent pediatric care for minority populations is essential [54,55], as in many busy practices, a lack of time for in-depth conversations limits opportunities to understand patients in all their complexities, including culture-related differences [56]. In terms of cultural differences, the risks for understanding problems and conflicts increase, which in combination with time pressure, may negatively influence the provider-patient relationship. The study shows that nurses, doulas, and mothers search and show images and films from the internet for illustration, as well as to substitute or complement verbal messages when culturally sensitive topics requiring complex and time-consuming explanations are discussed. This study confirms that pictorial information used for supporting interactions with people having limited or no knowledge of language (eg, newly arrived refugees in neonatal care and migrant workers) [42,57] is beneficial for managing communicative challenges.

The study participants were concerned about spending time searching for images and films on the internet. This finding, although preliminary, suggests that the development of targeted multimodal digital support, including pictorial and video resources, for the issues mentioned by the participants, such as nutrition, child bonding strategies, and safety, can potentially save time from “googling” [39] and make interactions more efficient. Further, the availability and accessibility of internet resources provide opportunities for accessing information after interactions and sharing it with other family members not present during the interactions. For example, fathers who have to work can still obtain information, which contributes to involving them in child care and to their integration in the host society [58].

Despite its exploratory nature, the results of this study indicate that ICT, including telephones, smartphones, computers, and the internet, provides opportunities for mitigating communication challenges and allows migrants to become actively involved in child care. Despite its limitations, this study adds to our knowledge of the use of ICT in pediatric encounters. However, further research with nurses, doulas, and migrant mothers to better understand their experiences of using ICT in pediatric encounters is needed. In addition, to obtain an in-depth understanding of the cultural differences for specific cultural groups, evaluations involving the perspectives of mothers and doulas from different cultural backgrounds are necessary.

### Conclusions

Considering the limited research available, this study is an important contribution to literature regarding the use of ICT by doulas and nurses in intercultural pediatric care. Taken together, the findings of this study show the complexity of providing culturally competent care. The research highlights that there is a need to embrace the advancement of technological artifacts and to gain more knowledge about the possibilities and limitations of the implementation of ICT and its role in managing language barriers and intercultural communication challenges, which are becoming increasingly relevant in a global society. Advancements in intelligent translation algorithms are important for increasing translation reliability and ensuring mutual understanding between patients and health care providers. The research contributes to theory by providing multidisciplinary findings in multiple research fields, such as information technology, mobile-assisted language learning, and intercultural health care communication.

### Limitations and Future Research

With regard to study limitations, more work needs to be done to determine how ICT can be adapted to the needs of professionals and users in the different sectors of health care. Further, the present article reflects the perspectives of a limited number of nurses and doulas. Finally, future research should focus on the perspectives of migrant parents.

## Abbreviations

CHC: child health care
ICT: information and communications technology
